# Determinants of health check attendance in adults: findings from the cross-sectional German Health Update (GEDA) study

**DOI:** 10.1186/1471-2458-14-913

**Published:** 2014-09-04

**Authors:** Jens Hoebel, Anne Starker, Susanne Jordan, Matthias Richter, Thomas Lampert

**Affiliations:** Department of Epidemiology and Health Monitoring, Robert Koch Institute, General-Pape-Straße 62-66, 12101 Berlin, Germany; Institute of Medical Sociology, Martin Luther University Halle-Wittenberg, Magdeburger Straße 8, 06112 Halle (Saale), Germany

**Keywords:** Health check, Cardiovascular diseases, Early diagnosis, Socioeconomic status, Secondary prevention, Screening

## Abstract

**Background:**

In Germany, adult health checks are carried out in the primary care setting for early detection of chronic conditions, such as cardiovascular diseases, diabetes, and kidney disease. This study aims to examine the social, behavioural, and health-related determinants of health check attendance among eligible adults in Germany.

**Methods:**

Data were derived from the cross-sectional German Health Update (GEDA) study, a national health survey among adults in Germany carried out by the Robert Koch Institute. Analyses were restricted to respondents with statutory health insurance aged 35 years or older (n = 26,555). Logistic regression models were fitted to estimate associations between health check attendance and factors selected on the basis of Andersen’s Behavioral Model of Health Services Use.

**Results:**

After mutual adjustment, higher health check attendance was associated with a higher age, higher socioeconomic status, being married, stronger social support, physical activity, non-smoking, greater fruit and vegetable consumption, and higher use of outpatient care in both sexes. In women, higher attendance was related to alcohol consumption and having company health insurance (BKK) after multiple adjustment. In men, higher attendance was associated with better self-rated health after adjusting for all other factors.

**Conclusions:**

The findings of this study suggest that people with an unfavourable risk factor profile, such as socioeconomically disadvantaged groups, smokers, physically inactive people, and persons with a low fruit and vegetable intake, are less likely to have health checks than those with a more favourable risk profile. Health checks carried out in the primary care setting should be evaluated for their effects on population health and health inequality.

## Background

In several health care systems of developed countries, general health checks are carried out for primary and secondary prevention of cardiovascular diseases, diabetes mellitus, and other chronic diseases [1]. One aim of general health checks is to lower the incidence of chronic diseases by reducing important risk factors for the purpose of primary prevention at the population level [2]. For secondary preventive purposes, health checks are aimed at detecting common diseases in an precursor or early stage in order to prevent the disease progression or chronification [3, 4]. An essential requirement for the success of such population-based prevention efforts is the participation of groups particularly vulnerable to chronic disease.

Numerous studies have shown that socioeconomically disadvantaged people are particularly vulnerable to cardiovascular diseases and diabetes because the prevalence and incidence of these conditions increase with lower socioeconomic status [5–9]. This might be largely due to the fact that important risk factors, such as obesity, physical inactivity, smoking, and psychosocial stress, are more prevalent in lower than in higher socioeconomic groups [10–13]. Therefore, socioeconomically disadvantaged people should be a key target group for interventions designed to reduce cardiovascular disease, diabetes, and respective risk factors.

Previous research has shown that the use of prevention services is lower among socioeconomically disadvantaged groups than among those who are better off [1, 14–22]. Furthermore, previous studies have suggested lower attendance rates among groups with an increased disease risk, such as smokers and physically inactive or obese people, even after adjusting for socioeconomic factors [15, 20, 23, 24]. These findings underline the challenge of tailoring population-based prevention programmes to specific vulnerable groups.

The German health insurance system is characterised by a dual system of statutory and private health insurance. More than 85% of the entire population in Germany is covered by the statutory health insurance. Compared to those with private health insurance, people with statutory health insurance are more often women and have a lower socioeconomic status [25]. According to the German Social Code (SGB V), people covered by statutory health insurance are eligible to participate in biennial health checks from the age of 35 years without any costs. These medical health checks are carried out in the primary care setting and are particularly aimed at the early detection of cardiovascular diseases, diabetes mellitus, kidney disease, and associated risk factors. Besides anamnesis and a physical examination, laboratory tests of blood and urine are carried out followed by medical counselling. Previous studies have suggested that approximately one in two eligible men and women in Germany attend this health check within 2 years [4, 26, 27]. Existing evidence on socioeconomic differences in attendance behaviour is inconsistent because some studies have indicated socioeconomic disparities and others have not [4, 26, 28–30]. To the best of our knowledge, associations between behavioural risk factors for cardiovascular disease or diabetes and attendance at the statutory health check in the German general population have not been investigated yet. A survey of primary care patients in a southern region of Germany showed that health-check participants were less likely to be smokers than non-participants [30]. Evidence from other countries also suggests that people with an unhealthy lifestyle, such as smoking or unhealthy diets, are less likely to present for a health check than those with a healthier lifestyle [1].

The present study aimed to examine the determinants of health check attendance among eligible adults in Germany. We used the Behavioral Model of Health Services Use developed by Andersen [31] as the conceptual framework guiding our investigation of health-seeking behaviour. This model is increasingly being used in research on preventive and curative health care use [3, 32]. Therefore, by referring to this model, we aimed at providing evidence which is comparable with previous findings on factors influencing the use of health services and prevention measures. According to the Andersen model, health care use is principally influenced by three major factor categories: predisposing, enabling, and need factors [31–33]. Predisposing factors influence the use of health services primarily indirectly, and consist of sociodemographic characteristics, such as age, gender/sex, socioeconomic or marital status, as well as psychological characteristics, such as health beliefs. Enabling factors encompass individual and contextual resources, which facilitate or impede the use of health services (e.g., type of health insurance or travel times arising from community structures). Need factors have a more direct effect on health services use and can be differentiated into perceived and evaluated components. Perceived need refers to how people view their own health status, and how they experience symptoms or worry about health and illness. Consequently, perceived need is a subjective judgement about the importance of care-seeking that is usually formed before consulting a health professional. In contrast, evaluated need represents a professional assessment of people’s need for medical care that proceeds after patients’ presentation to a health care provider [31].

In this study, three questions were investigated:

1. What are the social determinants of attendance at health checks among men and women in Germany?
2. What are the behavioural and health-related determinants of health check attendance?
3. Do determinants of health check attendance vary between men and women?

## Methods

### Study design and study population

Data were derived from the cross-sectional “German Health Update” (GEDA), a national telephone health interview survey among adults in Germany. The GEDA study is part of the nationwide Health Monitoring System administered by the Robert Koch Institute, Berlin. The Robert Koch Institute is a federal institution within the portfolio of the German Federal Ministry of Health responsible for disease control and prevention. The aim of the regularly conducted GEDA surveys is to provide current data on population health, health determinants, and use of health services for national and European health reporting systems, health policies, and public health research.

Data from the consecutive GEDA waves 2009 and 2010 were pooled to increase the statistical power of the analyses. Both survey waves were based on a two-stage sampling procedure. First, random samples of telephone numbers from the German fixed-line network were generated using the Gabler–Häder method, which assured that households without registered telephone numbers were included in the sample [34]. Second, the “last birthday method” was applied for random selection of respondents within a contacted household. The adult member of the household who had the last birthday or whose birthday was on the day of contact to the household was selected for the sample. A total of 43,312 respondents aged 18 to 100 years completed the survey from July 2008 to June 2009 (GEDA 2009) and from September 2009 to July 2010 (GEDA 2010). According to the internationally used Standard Definitions of outcome rates for surveys [35], the “Response Rate 3” was 29.1% in GEDA 2009 and 28.9% in GEDA 2010. The cooperation rate based on all contacted target subjects was 51.2% in GEDA 2009 and 55.8% in GEDA 2010. As the literature suggests that there is no clear relation between response rate and representativeness of response [36], a low response does not necessarily lead to strong selection or weak external validity. The current analyses were restricted to study participants with statutory health insurance aged 35 years and older (n = 26,555). Their sociodemographic characteristics are shown in Table 1.

**Table 1:** **Characteristics of the study participants with statutory health insurance aged 35 years or older**

	Men (n = 10 406)			Women (n = 16 149)		
	n	%^1^	%^2^	n	%^1^	%^2^
**Age**						
35–44 years	2816	27.1	26.0	4591	28.4	22.8
45–54 years	2747	26.4	25.9	4313	26.7	23.0
55–64 years	2082	20.0	18.8	3203	19.8	17.8
65–74 years	2004	19.3	20.6	2748	17.0	22.9
75+ years	757	7.3	8.8	1294	8.0	13.5
**Socioeconomic status**						
Low	1205	11.6	20.5	2034	12.6	24.2
Medium	5826	56.0	61.2	10180	63.0	62.6
High	3291	31.6	18.3	3893	24.1	13.2
Missing	84	0.8	–	42	0.3	–
**Marital status**						
Single	1555	14.9	12.0	1704	10.6	6.9
Separated/divorced/widowed	1786	17.2	13.4	5089	31.5	29.5
Married and living together	7046	67.7	74.6	9337	57.8	63.6
Missing	19	0.2	–	19	0.1	–

^1^Percentage of the sample; ^2^weighted percentage (weighting factors were used to account for the sampling design and to adjust for distribution of the sample by sex, age, education, and region to match the German population).

Data were collected by means of a standardised computer-assisted telephone interview (CATI). Besides information on health status, health determinants, and use of health services, data on sociodemographic characteristics were gathered in the survey. Interviews took an average of approximately 30 minutes. The study was approved by The Federal Commissioner for Data Protection and Freedom of Information, and verbal informed consent was obtained from all of the participants in advance.

### Health check attendance

Self-reported attendance at health checks was the outcome measure of this study. Respondents with an age of 35 years or older were initially asked whether they had ever attended the health check recommended by the statutory health insurance funds (“yes”, “no”, and “not sure”). Those persons who answered “yes” were asked a second question on attendance in the past 2 years (“yes” or “no”). We created a binary variable indicating health check attendance in the past 2 years (yes = 1, no = 0).

### Predisposing factors

Independent variables were selected by referring to the Behavioral Model of Health Services Use [31]. Predisposing factors encompassed age (35–44, 45–54, 55–64, 65–74, and 75+ years), socioeconomic status, and marital status. Socioeconomic status was measured with an additive multidimensional index based on school and vocational education, occupational status, and net equivalent household income [37]. This index was classified as low (quintile 1), medium (quintiles 2–4), and high (quintile 5) by quintiles of the whole population. A more detailed description of the socioeconomic status index, its sub-dimensions, and its calculation can be found elsewhere [37]. Marital status was grouped as married and living together, separated/divorced/widowed, and single.

### Enabling factors

In Germany, every person covered by statutory health insurance aged 35 years or older is eligible to have a biennial health check. However, the type of statutory health insurance was used in this study to consider possible differences in sickness funds’ strategies to inform their insurants about this early detection measure. We differentiated between local health insurance funds (Allgemeine Ortskrankenkasse, AOK), alternative health insurance funds (Ersatzkassen, EK), company health insurance funds (Betriebskrankenkassen, BKK), and others. The Oslo Three-Item Social Support Scale (OSS-3) was used to measure social support, which was assumed to be another factor enabling people to use health services. The OSS-3 sum score was classified as poor (3–8), intermediate (9–11), and strong (12–14) social support [38]. The type of residential area was considered to refer to the population density of the area in which the study participants lived. Based on classification by the German Federal Institute for Research on Building, Urban Affairs and Spatial Development, we assigned administrative districts to four types of areas: metropolitan, urban, rural with concentration signs, as well as rural and sparsely populated.

### Need factors

We used information on behavioural risk factors for cardiovascular diseases and diabetes, self-perceived health, and use of outpatient care to assess respondents’ need for health checks. By this means, perceived and evaluated need components were considered. Obesity was assessed by body mass index (BMI) using the World Health Organization criteria (BMI ≥ 30 kg/m^2^) [39]. BMI was calculated using self-reported height and weight. Physical activity was ascertained with two questions on the number of days per week and minutes per day of physical activity, which was defined as starting to sweat and getting out of breath. We classified physical activity as active (≥150 minutes/week), slightly active (<150 minutes/week), and not active at all (no weekly physical activity). We assessed respondents’ smoking status with one question on their smoking behaviour (current smokers, ex-smokers, and never-smokers). Alcohol consumption was measured using the Alcohol Use Disorders Identification Test–Consumption (AUDIT–C) [40]. According to internationally used cut points [41], the AUDIT–C sum score (0–12) was classified as hazardous consumption (men: ≥5, women: ≥4), moderate consumption (men: 1–4, women: 1–3), and never-drinking (0). We considered fruit and vegetable consumption in the analysis because fruit and vegetable intake is inversely associated with the risk of cardiovascular diseases [42, 43]. Respondents were asked for the number of fruit and vegetable portions they take in daily. We categorised the number of portions per day as low (quintile 1), moderate (quintiles 2–4), and high (quintile 5). Self-rated health was measured on a five-point scale and dichotomised as very good/good or fair/poor/very poor. Use of outpatient care was assessed by the number of resident physician visits during the past 12 months, and was classified as low (quintile 1), medium (quintiles 2–4), and high (quintile 5).

### Statistical analysis

Health check attendance rates were calculated by predisposing, enabling, and need factors. We used Pearson’s χ^2^-test and 95% confidence intervals (CIs) to examine statistically significant differences. Logistic regression models were fitted to estimate adjusted odds ratios (ORs) of attendance in the past 2 years with 95% CIs and p-values. In the first step, we computed age-adjusted ORs of attendance by each of the above-described factors in separate logistic regression models. In the second step, we used a full model containing all predisposing, enabling, and need factors as described above. All analyses were conducted separately for men and women to identify sex-specific factors associated with health check attendance and to prevent potential gender bias. Weighting factors were used to account for the sampling design and to adjust for distribution of the sample by sex, age, education, and region to match the German population. All statistical analyses were performed using STATA 12.0 survey data procedures.

## Results

### Bivariate analysis

The 2-year health check attendance rate was 50.8% in men and 49.8% in women. Attendance was associated with each of the predisposing factors (Table 2). In men and women, attendance increased with age up to 74 years, but declined thereafter. Men and women with low socioeconomic status were less likely to use the health check within 2 years than those with medium and high status. Single persons attended less often than married or formerly married people. Most enabling factors were bivariately associated with the uptake of health checks. Attendance increased with stronger social support and varied across statutory health insurance funds. The type of residential area was not related to attendance behaviour. Moreover, attendance at health checks was associated with each of the need factors considered. Only physical activity and alcohol consumption were not found to be related to the attendance behaviour of men.

**Table 2:** **Health check attendance in the past 2 years by predisposing, enabling, and need factors**

	Men		Women	
	% (95% CI)	p-value	% (95% CI)	p-value
**Total**	50.8 (49.6–52.0)		49.8 (48.8–50.9)	
***Predisposing factors***				
**Age**				
35–44 years	32.7 (30.5–34.9)		36.1 (34.4–37.8)	
45–54 years	48.8 (46.5–51.2)		48.5 (46.6–50.4)	
55–64 years	61.0 (58.3–63.6)		54.7 (52.5–56.9)	
65–74 years	64.7 (61.8–67.4)		59.7 (57.2–62.2)	
75+ years	55.9 (51.2–60.6)	<0.001	52.4 (48.8–56.0)	<0.001
**Socioeconomic status**				
Low	45.0 (41.7–48.4)		45.5 (42.9–48.3)	
Medium	51.1 (49.5–52.6)		51.3 (50.1–52.6)	
High	55.9 (53.9–57.9)	<0.001	50.6 (48.7–52.5)	<0.001
**Marital status**				
Single	36.0 (32.9–39.3)		40.2 (37.2–43.2)	
Separated/divorced/widowed	47.2 (44.0–50.4)		51.4 (49.5–53.3)	
Married and living together	53.8 (52.4–55.3)	<0.001	50.2 (48.8–51.5)	<0.001
***Enabling factors***				
**Statutory health insurance**				
AOK	47.8 (45.4–50.3)		47.3 (45.2–49.4)	
EK	55.0 (53.1–56.9)		51.3 (49.9–52.8)	
BKK	50.1 (47.5–52.7)		50.6 (48.1–53.0)	
Other	47.8 (44.1–51.5)	<0.001	50.4 (46.8–53.9)	<0.05
**Social support**				
Poor	43.6 (40.4–46.8)		45.5 (42.9–48.2)	
Intermediate	51.9 (50.1–53.6)		50.5 (49.0–52.0)	
Strong	54.0 (51.8–56.1)	<0.001	51.9 (50.1–53.7)	<0.001
**Type of residential area**				
Rural and sparsely populated	50.2 (47.1–53.2)		48.0 (45.3–50.6)	
Rural with concentration signs	52.3 (49.3–55.3)		49.6 (46.9–52.2)	
Urban	52.5 (50.5–54.5)		51.0 (49.3–52.7)	
Metropolitan	48.4 (46.0–50.8)	n.s.	49.7 (47.8–51.6)	n.s.
***Need factors***				
**Obesity**				
No	50.2 (48.8–51.6)		49.6 (48.4–50.8)	
Yes	53.6 (50.7–56.5)	<0.05	52.7 (50.0–55.3)	<0.05
**Physical activity**				
Active	52.0 (50.1–54.0)		51.5 (49.8–53.2)	
Slightly active	50.5 (48.5–52.5)		51.5 (49.8–53.2)	
Not active at all	49.1 (46.4–51.7)	n.s.	46.3 (44.2–48.5)	<0.001
**Smoking**				
Non-smoker	55.2 (53.7–56.7)		52.9 (51.7–54.2)	
Smoker	41.2 (39.0–43.4)	<0.001	40.0 (38.1–41.9)	<0.001
**Alcohol consumption**				
Never	48.3 (44.9–51.9)		45.0 (42.7–47.2)	
Moderate	51.9 (50.2–53.5)		51.9 (50.5–53.3)	
Hazardous	50.0 (47.8–52.3)	n.s.	51.1 (48.8–53.3)	<0.001
**Fruit/vegetable consumption**				
Low	42.7 (40.3–45.0)		38.9 (36.3–41.5)	
Medium	53.2 (51.6–54.8)		51.0 (49.6–52.4)	
High	59.6 (56.1–63.0)	<0.001	54.6 (52.6–56.7)	<0.001
**Self-rated health**				
Very good/good	49.6 (48.1–51.1)		48.1 (46.8–49.3)	
Fair/poor/very poor	53.1 (50.8–55.3)	<0.05	52.8 (50.9–54.6)	<0.001
**Outpatient care use**				
Low	35.1 (33.0–37.2)		33.2 (31.2–35.2)	
Medium	57.2 (55.5–58.9)		54.1 (52.7–55.4)	
High	60.2 (57.0–63.3)	<0.001	58.3 (55.7–60.8)	<0.001

CI = confidence interval, n.s. = not significant (p > 0.05), AOK = local health insurance funds, EK = alternative health insurance funds, BKK = company health insurance funds.

### Age-adjusted analysis

After adjusting for age, the odds of attendance increased with higher socioeconomic status in men and women (Table 3). Married people living with their spouse had a greater odds of attendance than single men and women. Persons who were separated, divorced, or widowed were not found to have higher odds of health check use than single persons when age was controlled for. The variation of attendance across statutory health insurance funds and the increase in attendance with stronger social support remained significant after adjusting for age. Women living in urban areas had higher odds of using health checks than those living in sparsely populated areas; this association was not found in men. The relationships between need factors and health check attendance were altered by adjusting for age in that obesity and self-rated health were no longer associated with attendance. Physically inactive people and smokers had lower odds of using health checks than those who were physically active and non-smokers, respectively. In men and women, moderate alcohol consumption, greater fruit and vegetable intake, and higher use of outpatient care were related to increased odds of attendance when age was controlled for. In women, hazardous alcohol consumption was related to higher attendance compared with no alcohol use.

**Table 3:** **Odds ratios of health check attendance in the past 2 years by predisposing, enabling, and need factors**

	Men		Women	
	Age-adjusted OR (95% CI)	Multiple-adjusted OR (95% CI)	Age-adjusted OR (95% CI)	Multiple-adjusted OR (95% CI)
***Predisposing factors***				
**Age**				
35–44 years	–	1.00 (Ref.)	–	1.00 (Ref.)
45–54 years	–	2.03 (1.75-2.36)***	–	1.63 (1.45-1.83)***
55–64 years	–	3.21 (2.71-3.80)***	–	2.03 (1.78-2.31)***
65–74 years	–	3.33 (2.76-4.01)***	–	2.54 (2.18-2.96)***
75+ years	–	2.52 (1.92-3.30)***	–	2.26 (1.82-2.81)***
**Socioeconomic status**				
Low	1.00 (Ref.)	1.00 (Ref.)	1.00 (Ref.)	1.00 (Ref.)
Medium	1.34 (1.15–1.57)***	1.22 (1.02-1.45)*	1.42 (1.26–1.61)***	1.35 (1.16-1.56)***
High	1.70 (1.44–2.00)***	1.43 (1.18-1.74)***	1.56 (1.37–1.79)***	1.34 (1.14-1.59)***
**Marital status**				
Single	1.00 (Ref.)	1.00 (Ref.)	1.00 (Ref.)	1.00 (Ref.)
Separated/divorced/widowed	1.07 (0.87–1.31)	1.08 (0.87-1.35)	1.16 (0.99–1.35)	1.19 (1.00-1.41)
Married and living together	1.55 (1.32–1.82)***	1.44 (1.21-1.72)***	1.26 (1.09–1.45)**	1.17 (1.00-1.36)*
***Enabling factors***				
**Statutory health insurance**				
AOK	1.00 (Ref.)	1.00 (Ref.)	1.00 (Ref.)	1.00 (Ref.)
EK	1.34 (1.18–1.53)***	1.06 (0.91-1.23)	1.21 (1.09–1.34)***	1.04 (0.92-1.17)
BKK	1.26 (1.09–1.47)**	1.09 (0.92-1.29)	1.28 (1.12–1.47)***	1.25 (1.08-1.45)**
Other	1.11 (0.92–1.33)	1.00 (0.82-1.23)	1.17 (0.98–1.38)	1.10 (0.91-1.32)
**Social support**				
Poor	1.00 (Ref.)	1.00 (Ref.)	1.00 (Ref.)	1.00 (Ref.)
Intermediate	1.49 (1.28–1.74)***	1.33 (1.13-1.58)***	1.28 (1.13–1.45)***	1.15 (1.00-1.32)
Strong	1.73 (1.47–2.04)***	1.49 (1.24-1.78)***	1.41 (1.24–1.61)***	1.24 (1.07-1.43)**
**Type of residential area**				
Rural and sparsely populated	1.00 (Ref.)	1.00 (Ref.)	1.00 (Ref.)	1.00 (Ref.)
Rural with concentration signs	1.10 (0.92-1.31)	1.17 (0.96-1.42)	1.07 (0.92-1.21)	1.05 (0.89-1.24)
Urban	1.14 (0.98–1.33)	1.18 (0.99-1.39)	1.15 (1.01–1.30)*	1.13 (0.98-1.30)
Metropolitan	0.94 (0.80–1.11)	1.09 (0.91-1.30)	1.06 (0.92–1.21)	1.08 (0.93-1.26)
***Need factors***				
**Obesity**				
No	1.00 (Ref.)	1.00 (Ref.)	1.00 (Ref.)	1.00 (Ref.)
Yes	1.08 (0.95–1.24)	1.03 (0.88-1.19)	1.02 (0.91–1.15)	1.08 (0.94-1.23)
**Physical activity**				
Active	1.00 (Ref.)	1.00 (Ref.)	1.00 (Ref.)	1.00 (Ref.)
Slightly active	0.92 (0.81–1.03)	0.87 (0.77-0.99)*	1.01 (0.92–1.11)	0.94 (0.85-1.04)
Not active at all	0.70 (0.61–0.81)***	0.79 (0.68-0.93)**	0.70 (0.62–0.79)***	0.78 (0.69-0.89)***
**Smoking**				
Non-smoker	1.00 (Ref.)	1.00 (Ref.)	1.00 (Ref.)	1.00 (Ref.)
Smoker	0.68 (0.61–0.76)***	0.78 (0.68-0.88)***	0.67 (0.61–0.74)***	0.77 (0.69-0.85)***
**Alcohol consumption**				
Never	1.00 (Ref.)	1.00 (Ref.)	1.00 (Ref.)	1.00 (Ref.)
Moderate	1.21 (1.03–1.41)*	1.07 (0.90-1.28)	1.43 (1.28–1.60)***	1.30 (1.15-1.48)***
Hazardous	1.12 (0.95–1.33)	1.05 (0.86-1.27)	1.38 (1.21–1.58)***	1.29 (1.11-1.50)***
**Fruit/vegetable consumption**				
Low	1.00 (Ref.)	1.00 (Ref.)	1.00 (Ref.)	1.00 (Ref.)
Medium	1.39 (1.23–1.57)***	1.19 (1.04-1.36)*	1.51 (1.34–1.71)***	1.39 (1.21-1.59)***
High	1.69 (1.41–2.01)***	1.36 (1.12-1.65)**	1.70 (1.48–1.96) ***	1.43 (1.23-1.68)***
**Self-rated health**				
Very good/good	1.00 (Ref.)	1.00 (Ref.)	1.00 (Ref.)	1.00 (Ref.)
Fair/poor/very poor	0.91 (0.81–1.02)	0.82 (0.71-0.94)**	1.03 (0.93–1.13)	0.94 (0.83-1.05)
**Outpatient care use**				
Low	1.00 (Ref.)	1.00 (Ref.)	1.00 (Ref.)	1.00 (Ref.)
Medium	2.25 (1.99–2.54)***	2.26 (1.98-2.58)***	2.26 (2.03–2.52)***	2.16 (1.93-2.42)***
High	2.41 (2.04–2.85)***	2.86 (2.35-3.47)***	2.55 (2.22–2.93)***	2.59 (2.21-3.04)***

*p < 0.05. **p < 0.01. ***p < 0.001. OR = odds ratio. CI = confidence interval. Ref. = reference category. AOK = local health insurance funds. EK = alternative health insurance funds. BKK = company health insurance funds.

### Multivariate analysis

After mutual adjustment for all predisposing, enabling, and need factors (Table 3), men and women aged 45 years and older had an increased odds of attendance compared with those aged less than 45 years. The association between higher socioeconomic status and health check attendance remained after multivariate adjustment, and married people who lived with their spouse had higher odds of attendance than single persons. In women, having statutory company health insurance (BKK) was associated with an increased odds of using health checks compared with those having statutory local health insurance (AOK). Social support remained as a predictor of health check attendance, while the type of residential area was not related to attendance behaviour after multiple adjustment. Obesity did not predict attendance behaviour in men or women. Physical inactivity and smoking remained associated with a lower odds of attendance in both sexes. Alcohol consumption persisted as a predictor of using health checks only in women. In men, poorer self-rated health was related to lower attendance. Higher fruit and vegetable consumption, as well as higher use of outpatient care, remained associated with a higher odds of health check attendance in men and women after adjusting for all factors.

## Discussion

The aim of this study was to examine the social, behavioural, and health-related determinants of attending health checks among men and women in Germany. Our findings indicated that health check attendance was associated with higher age, higher socioeconomic status, being married, stronger social support, physical activity, non-smoking, greater fruit and vegetable consumption, and higher outpatient care use in men and women after mutual adjustment. Differences between men and women were observed with regard to type of health insurance fund, alcohol consumption, and self-rated health. Women’s uptake of health checks was found to be related to alcohol consumption and having statutory company health insurance (BKK), while attendance of men was associated with better self-rated health after multivariate adjustment.

### Comparison with previous research

The findings of the GEDA study are in line with previous studies from Germany and other countries showing that younger people are less likely to participate in health checks than older people [1, 29, 30, 44]. However, some studies did not find any relationship between age and participation [24, 45]. Moreover, our findings support evidence from previous German studies, which found that the uptake of prevention services varies with socioeconomic factors. Janßen et al. [17] and Klein et al. [19] conducted systematic literature reviews on socioeconomic differences in the use of (preventive) health services in Germany. Both reviews showed that socioeconomically disadvantaged groups are less likely to use preventive services than socioeconomically privileged groups. This relationship has also been found in other countries and different health care systems [1, 15, 16, 20, 21, 24, 46]. However, the above-mentioned reviews [17, 19] also showed that the pertinent evidence from Germany is more consistent for cancer screening programmes, check-ups for children, or primary prevention measures than for the statutory health check for adults [18, 26, 28–30]. This finding could be explained by methodological issues, such as the use of different indicators of socioeconomic status, or the fact that, in some studies on health check attendance in Germany, people with private health insurance had not been excluded from the analysis [26, 29]. The present findings add to previous evidence on social inequalities in health check attendance derived from the German GEDA study [4] that the socioeconomic patterning of attendance behaviour remains after adjustment for enabling factors, which were not considered in the preceding analyses.

Our findings are consistent with previous studies, which showed that married people are more likely to use preventive measures than single persons [1, 46, 47]. A possible explanation for this finding could be that living together with a partner is associated with a mutual “health monitoring” [48], which could result in increased attention to health or symptoms, and a higher participation in prevention measures.

Previous research has suggested that social support is a promoter for the uptake of early detection measures [14, 49, 50], which is also supported by our findings. Social relations might facilitate the use of secondary prevention services (e.g., friends taking people to the doctor [51] or neighbours undertaking tasks of daily living, such as caring for children or relatives, when people visit a health professional). Additionally, the prospect of not being alone after obtaining a diagnosis could reduce concerns about participating in a medical check-up. These assumptions should be investigated prospectively. While the present study considered general social support, future studies on health care-seeking behaviour could also assess specific support with reference to the use of health services.

Our findings are also consistent with previous studies on the role of need factors as determinants of health check attendance. Dryden et al. [1] showed in a literature review that non-attenders have a less favourable cardiovascular risk factor profile than attenders, especially smokers are less likely to attend health checks than non-smokers. Moreover, other studies have shown a facilitating effect of regular doctor visits on the use of early detection measures [15, 47, 52, 53]. This finding is consistent with our study, which showed a strong association between higher use of outpatient care and the uptake of health checks. Frequent consulters of primary care are probably more likely to receive invitations to secondary prevention services from health professionals than people outside the healthcare system. However, Dryden and colleagues [1] have also reported that ongoing contact with physicians might be a reason for not attending health checks. Because of this inconsistency between studies, further research on the relation between primary care use and health check attendance is required.

The present findings also support evidence suggesting that determinants of care-seeking behaviour differ between men and women, apparently in close connection with societal gender roles [54, 55]. Although there is a growing body of studies showing differences in care-seeking behaviour between men and women, the number of studies explicitly analysing the sex or gender differences in health care utilisation is still low [54]. In future research, a stronger distinction between sex as a biological and gender as a social category may help to better understand these differences.

The observed differences in women's health check attendance by the type of statutory health insurance may reflect potential differences in the way health insurance funds inform their insurants about health checks or encourage them to use this service. Possibly, information provided by company health insurance funds is more appealing to women than men.

### Effects of health checks

Future studies also need to examine the effects of health checks on population health. A systematic review on the benefits and harms of general health checks indicated that such preventive measures do not reduce morbidity or mortality from disease, although the number of new diagnoses has increased [56]. Potential harms of health checks, such as overdiagnosis or negative psychological consequences for patients, have rarely been studied. The authors of the systematic review mentioned above acknowledged that many trials had methodological problems and most of the trials were old [56]. The oldest study was from the 1960s and the most recent was from the 2000s. Within this time span, there have been many changes in medical practice [57]. Therefore, the transferability of the results to current settings might be limited. Another systematic review and meta-analysis showed that health checks carried out in general practice are associated with statistically significant, but clinically small, improvements in surrogate outcomes, such as blood pressure and BMI [58]. Accordingly, beneficial effects of health checks are still controversial [59, 60]. To the best of our knowledge, no evidence is available yet concerning the effects of the statutory health check for adults in Germany. Currently, there is a lively and controversial debate on the benefits and harms of cancer screening programmes in Germany. However, the potential effects of the statutory health check seem to be barely on the agenda, although this early detection measure also may potentially lead to overdiagnosis and overtreatment. Therefore, population-based measures for early diagnosis, such as the statutory health check, should be supervised scientifically to monitor the expected effects on population health, as well as potential harms for the participants and the health system.

### Methodological considerations

The strengths of this study include the use of up-to-date data from large national samples and the wide range of explanatory variables considered in the analyses. The large sample size also allowed separate analyses for men and women. Nevertheless, there are several limitations to our study. We used self-reported attendance at health checks as the outcome measure. Self-reports on the use of health checks may be biased by social desirability and by confusing other medical services with health checks. We accounted for the latter possibility by adding the category “not sure” to the response options (treated as missing data in the analysis). The low percentage of respondents who used this category (2.6%) indicated that potential bias due to uncertainty of respondents should be small. Moreover, the recall period of 2 years may have led to recall bias in the assessment of health check attendance. Social desirability might also have affected responses on questions regarding social support and health behaviour. This may have contributed to associations between these indicators and health check attendance [61].

Because of the cross-sectional nature of the study, no causal relationships could be established and there may have been potential bias related to reverse causality. If respondents who had attended a health check had a more favourable risk factor profile as a result of their health check participation, the effects of behavioural risk factors on attendance may have been overestimated. Furthermore, results may have been affected by selection bias due to underrepresentation of certain population groups in the sample, such as people with a migration background, because interviews were only conducted in German. For that reason, migration or ethnic background was not considered in the analyses. Other limitations arise from the selection and measurement of factors considered in the study. The type of residential area may only be a proxy measure for regional density of primary care physicians. This might have contributed to our finding that there was no regional variation in health check attendance. A study which examined the use of cancer screening in Germany, for instance, revealed that screening rates are higher in areas with higher physician density [62]. Psychological factors, such as health beliefs, and contextual factors, such as regional physician density, were not considered in the analysis. However, such factors are considered to determine health care use in the Behavioral Model of Health Services Use [31, 63]. The assessment of physical activity and its definition in the questionnaire may have been imprecise; weekly metabolic equivalents may have been a better indicator of physical activity. Moreover, non-behavioural need factors, such as family history of diseases or psychosocial stress, could not be controlled for. This may have led to residual or unmeasured confounding. Besides, it has to be considered that failing to see an association (beta error) may have been more likely in men than in women due to a lower number of men in the study population.

## Conclusions

Overall, the findings of this study suggest that population groups with a higher risk of adverse health, such as the socioeconomically disadvantaged, smokers, and physically inactive people, are less likely to attend health checks than their counterparts with a more favourable risk factor profile. Therefore, those who potentially could benefit most from secondary prevention measures appear to be particularly difficult to engage with medical health checks offered in the primary care setting. This should be taken into more account when designing and implementing secondary prevention programmes at the population level. As already stated in previous research [1, 44], differential uptake of health checks may lead to an exacerbation of health inequalities via the inverse care law, which states that the availability of good medical care tends to vary inversely with the need for it in the population served [64, 65]. Whereas this study focused on health check attendance, future research should also investigate how the quality and delivery of prevention services by health care providers vary with the characteristics of attenders, such as their socioeconomic status. The findings of the present study indicate that population-based prevention measures should be tailored more appropriately to the needs of socioeconomically less privileged groups and people with risk factors for cardiovascular diseases and diabetes. This underlines the need to develop and improve target-group-specific approaches for public health interventions. In Germany, there is still a lack of evidence on how to better engage vulnerable groups with prevention services [17]. This issue is an important challenge for future research. A higher participation of vulnerable groups might potentially contribute to achieve an effect on population health and, on the condition of beneficial effects, to reduce social inequalities in health.

## References

[1] Dryden R, Williams B, McCowan C, Themessl-Huber M (2012). What do we know about who does and does not attend general health checks? Findings from a narrative scoping review. BMC Public Health.

[2] Cochrane T, Davey R, Iqbal Z, Gidlow C, Kumar J, Chambers R, Mawby Y (2012). NHS health checks through general practice: randomised trial of population cardiovascular risk reduction. BMC Public Health.

[3] Janssen C, Swart E, Lengerke T von (2014). Health care utilization in germany - theory, methodology, and results.

[4] Hoebel J, Richter M, Lampert T (2013). Social status and participation in health checks in men and women in Germany - results from the German Health Update (GEDA), 2009 and 2010. Dtsch Arztebl Int.

[5] Agardh E, Allebeck P, Hallqvist J, Moradi T, Sidorchuk A (2011). Type 2 diabetes incidence and socio-economic position: a systematic review and meta-analysis. Int J Epidemiol.

[6] Demakakos P, Marmot M, Steptoe A (2012). Socioeconomic position and the incidence of type 2 diabetes: the ELSA study. Eur J Epidemiol.

[7] Gösswald A, Schienkiewitz A, Nowossadeck E, Busch MA (2013). Prevalence of myocardial infarction and coronary heart disease in adults aged 40–79 years in Germany: results of the German Health Interview and Examination Survey for Adults (DEGS1). Bundesgesundheitsblatt Gesundheitsforschung Gesundheitsschutz.

[8] Heidemann C, Du Y, Schubert I, Rathmann W, Scheidt-Nave C (2013). Prevalence and temporal trend of known diabetes mellitus: results of the German Health Interview and Examination Survey for Adults (DEGS1). Bundesgesundheitsblatt Gesundheitsforschung Gesundheitsschutz.

[9] Lampert T, Kroll LE, von der Lippe E, Muters S, Stolzenberg H (2013). Socioeconomic status and health: results of the German Health Interview and Examination Survey for Adults (DEGS1). Bundesgesundheitsblatt Gesundheitsforschung Gesundheitsschutz.

[10] Kuntz B, Lampert T (2010). Socioeconomic factors and obesity. Dtsch Arztebl Int.

[11] Lampert T (2010). Smoking, physical inactivity, and obesity: associations with social status. Dtsch Arztebl Int.

[12] Dragano N, Bobak M, Wege N, Peasey A, Verde PE, Kubinova R, Weyers S, Moebus S, Mohlenkamp S, Stang A, Erbel R, Jöckel K-H, Siegrist J, Pikhart H (2007). Neighbourhood socioeconomic status and cardiovascular risk factors: a multilevel analysis of nine cities in the Czech Republic and Germany. BMC Public Health.

[13] Siegrist J (2006). Was trägt Stressforschung zur Erklärung des sozialen Gradienten koronarer Herzkrankheiten bei? [What is the contribution of stress research towards explaining the social gradient of coronary heart disease?]. Dtsch Med Wochenschr.

[14] Bronner K, Mesters I, Weiss-Meilik A, Geva R, Rozner G, Strul H, Inbar M, Halpern Z, Kariv R (2013). Determinants of adherence to screening by colonoscopy in individuals with a family history of colorectal cancer. Patient Educ Couns.

[15] Brennenstuhl S, Fuller-Thomson E, Popova S (2010). Prevalence and factors associated with colorectal cancer screening in Canadian women. J Womens Health (Larchmt).

[16] Chiu BCH, Anderson JR, Corbin D (2005). Predictors of prostate cancer screening among health fair participants. Public Health.

[17] Janßen C, Sauter S, Kowalski C (2012). The influence of social determinants on the use of prevention and health promotion services: Results of a systematic literature review. Psycho-social Med.

[18] Jordan S, von der Lippe E (2013). Participation in health behaviour change programmes: results of the German Health Interview and Examination Survey for Adults (DEGS1). Bundesgesundheitsblatt Gesundheitsforschung Gesundheitsschutz.

[19] Klein J, Hofreuter-Gätgens K, Knesebeck O, Janssen C, Swart E, von Lengerke T (2014). Socioeconomic status and the utilization of health services in Germany: a systematic review. Health care utilization in germany - theory, methodology, and results.

[20] Lee K, Lim HT, Park SM (2010). Factors associated with use of breast cancer screening services by women aged greater than or equal to 40 years in Korea: The Third Korea National Health and Nutrition Examination Survey 2005 (KNHANES III). BMC Cancer.

[21] Stanley SL, King JB, Thomas CC, Richardson LC (2013). Factors associated with never being screened for colorectal cancer. J Community Health.

[22] Starker A, Sass AC (2013). Participation in cancer screening in Germany: results of the German Health Interview and Examination Survey for Adults (DEGS1). Bundesgesundheitsblatt Gesundheitsforschung Gesundheitsschutz.

[23] Rahman SMM, Dignan MB, Shelton BJ (2003). Factors influencing adherence to guidelines for screening mammography among women aged 40 years and older. Ethn Dis.

[24] Thorogood M, Coulter A, Jones L, Yudkin P, Muir J, Mant D (1993). Factors affecting response to an invitation to attend for a health check. J Epidemiol Community Health.

[25] Hoffmann F, Icks A (2012). Unterschiede in der Versichertenstruktur von Krankenkassen und deren Auswirkungen für die Versorgungsforschung: Ergebnisse des Bertelsmann-Gesundheitsmonitors [Structural Differences between Health Insurance Funds and their Impact on Health Services Research: Results from the Bertelsmann Health-Care Monitor]. Gesundheitswesen.

[26] Bergmann E, Kalcklosch M, Tiemann F (2003). Inanspruchnahme des Gesundheitswesens - Erste Ergebnisse des telefonischen Gesundheitssurveys 2003 [Public health care utilisation - initial results of the Telephone Health Survey 2003]. Bundesgesundheitsblatt Gesundheitsforschung Gesundheitsschutz.

[27] Central Research Institute of Ambulatory Health Care in Germany: *Utilization of statutory health check-up. Documentation of the examination findings arising from the statutory measures for the early detection according to Article 25 of the German (SGB - Sozialgesetzbuch) Code of Social Law V*. [http://www.gbe-bund.de]

[28] Freund T, Lekutat C, Schwantes U, Szecsenyi J, Joos S (2010). Gesundheitsuntersuchung und Impfstatus – Ergebnisse einer Patientenbefragung in deutschen Hausarztpraxen [Health screening and vaccination status – results from a patient survey in German general practices]. Gesundheitswesen.

[29] Richter M, Brand H, Rössler G (2002). Sozioökonomische Unterschiede in der Inanspruchnahme von Früherkennungsuntersuchungen und Maßnahmen der Gesundheitsförderung in NRW [Socio-economic differences in the utilisation of screening programmes and health promotion measures in North Rhine-Westphalia, Germany]. Gesundheitswesen.

[30] Sönnichsen AC, Sperling T, Donner-Banzhoff N, Baum E (2007). Unterschiede zwischen Teilnehmern und Nichtteilnehmern an der Gesundheitsuntersuchung [Differences between participants and non-participants in health screening]. Z Allg Med.

[31] Andersen RM (1995). Revisiting the behavioral model and access to medical care: does it matter?. J Health Soc Behav.

[32] Babitsch B, Gohl D, von Lengerke T (2012). Re-revisiting Andersen’s Behavioral Model of Health Services Use: a systematic review of studies from 1998–2011. Psycho-social Med.

[33] Lengerke T, Gohl D, Babitsch B, Janssen C, Swart E, von Lengerke T (2014). Re-revisiting the Behavioral Model of Health Care Utilization by Andersen: a review on theoretical advances and perspectives. Health care utilization in germany - theory, methodology, and results.

[34] Gabler S, Häder S (1999). Generierung von Telefonstichproben mit TelSuSa [Generating telephone samples using TelSuSa]. ZUMA-Nachrichten.

[35] American Association for Public Opinion Research (AAPOR) (2011). Standard definitions - final dispositions of case codes and outcome rates for surveys (revised 2011).

[36] Schouten B, Cobben F, Bethlehem J (2009). Indicators for the representativeness of survey response. Survey Methodol.

[37] Lampert T, Kroll LE, Müters S, Stolzenberg H (2013). Messung des soziookonomischen Status in der Studie “Gesundheit in Deutschland aktuell” (GEDA) [Measurement of the socioeconomic status within the German Health Update 2009 (GEDA)]. Bundesgesundheitsblatt Gesundheitsforschung Gesundheitsschutz.

[38] Kilpeläinen K, Arpo A, ECHIM Core Group (2008). European health indicators - development and initial implementation.

[39] World Health Organization (2000). Obesity - Preventing and managing the global epidemic.

[40] Bush K, Kivlahan DR, McDonell MB, Fihn SD, Bradley KA (1998). The AUDIT alcohol consumption questions (AUDIT-C): an effective brief screening test for problem drinking. Arch Intern Med.

[41] Reinert DF, Allen JP (2007). The Alcohol Use Disorders Identification Test: an update of research findings. Alcohol Clin Exp Res.

[42] Dauchet L, Amouyel P, Hercberg S, Dallongeville J (2006). Fruit and vegetable consumption and risk of coronary heart disease: a meta-analysis of cohort studies. J Nutr.

[43] He FJ, Nowson CA, MacGregor GA (2006). Fruit and vegetable consumption and stroke: meta-analysis of cohort studies. Lancet.

[44] Waller D, Agass M, Mant D, Coulter A, Fuller A, Jones L (1990). Health checks in general practice: another example of inverse care?. BMJ.

[45] Hsu HY, Gallinagh R (2001). The relationships between health beliefs and utilization of free health examinations in older people living in a community setting in Taiwan. J Adv Nurs.

[46] Martin-Lopez R, Hernandez-Barrera V, de Andres AL, Carrasco-Garrido P, de Miguel AG, Jimenez-Garcia R (2012). Trend in cervical cancer screening in Spain (2003–2009) and predictors of adherence. Eur J Cancer Prev.

[47] Guessous I, Dash C, Lapin P, Doroshenk M, Smith RA, Klabunde CN, National Colorectal Cancer Roundtable Screening Among the 65 Plus Task G (2010). Colorectal cancer screening barriers and facilitators in older persons. Prev Med.

[48] Winkelhake O, Mielck A, John J (1997). Einkommen, Gesundheit und Inanspruchnahme des Gesundheitswesens in Deutschland 1992 [Income, health, and use of health services in Germany 1992]. Soz Praventivmed.

[49] Mishra SI, DeForge B, Barnet B, Ntiri S, Grant L (2012). Social determinants of breast cancer screening in urban primary care practices: a community-engaged formative study. Womens Health Issues.

[50] Oh KM, Jacobsen KH (2014). Colorectal cancer screening among Korean Americans: a systematic review. J Community Health.

[51] Andersen RM, Janssen C, Swart E, von Lengerke T (2014). Foreword. Health care utilization in germany - theory, methodology, and results.

[52] Labeit A, Peinemann F, Baker R (2013). Utilisation of preventative health check-ups in the UK: findings from individual-level repeated cross-sectional data from 1992 to 2008. BMJ Open.

[53] Lee HY, Lundquist M, Ju E, Luo X, Townsend A (2011). Colorectal cancer screening disparities in Asian Americans and Pacific Islanders: which groups are most vulnerable?. Ethn Health.

[54] Babitsch B, Bormann C, Gohl D, Ciupitu-Plath CC, Janssen C, Swart E, von Lengerke T (2014). Gender and utilization of health care. Health care utilization in germany - theory, methodology, and results.

[55] Tedstone Doherty D, Kartalova-O'Doherty Y (2010). Gender and self-reported mental health problems: predictors of help seeking from a general practitioner. Br J Health Psychol.

[56] Krogsbøll LT, Jørgensen KJ, Grønhøj Larsen C, Gøtzsche PC (2012). General health checks in adults for reducing morbidity and mortality from disease. Cochrane Database Syst Rev.

[57] Prochazka AV, Caverly T (2013). General health checks in adults for reducing morbidity and mortality from disease: summary review of primary findings and conclusions. JAMA Int Med.

[58] Si S, Moss JR, Sullivan TR, Newton SS, Stocks NP (2014). Effectiveness of general practice-based health checks: a systematic review and meta-analysis. Br J Gen Pract.

[59] Lauritzen T, Sandbaek A, Borch-Johnsen K (2014). General health checks may work. BMJ.

[60] Mangin D (2014). Ethical issues related to health checks. BMJ.

[61] Podsakoff PM, MacKenzie SB, Lee JY, Podsakoff NP (2003). Common method biases in behavioral research: a critical review of the literature and recommended remedies. J Appl Psychol.

[62] Vogt V, Siegel M, Sundmacher L (2014). Examining regional variation in the use of cancer screening in Germany. Soc Sci Med.

[63] Andersen RM, Davidson PL, Andersen RM, Rice TH, Kominski GF (2007). Improving access to care in America: individual and contextual indicators. Changing the US health care system.

[64] Hart JT (1971). The inverse care law. Lancet.

[65] Watt G (2002). The inverse care law today. Lancet.

[CR66] The pre-publication history for this paper can be accessed here:http://www.biomedcentral.com/1471-2458/14/913/prepub

